# Causality analysis of toxicological mechanisms in networked systems such as adverse outcome pathway networks

**DOI:** 10.1007/s00204-026-04386-z

**Published:** 2026-06-27

**Authors:** Thomas Hartung, Karolina Kopańska, Alexandra Maertens, Paul Whaley, Sebastian Hoffmann

**Affiliations:** 1 Center for Alternatives to Animal Testing (CAAT), Bloomberg School of Public Health and Whiting School of Engineering, Johns Hopkins University, Baltimore, MD USA; 2 CAAT-Europe, University of Konstanz, Konstanz, Germany; 3 Doerenkamp-Zbinden Chair for Evidence-Based Toxicology, Baltimore, MD USA; 4 CAATevents, Solingen, Germany; 5 Evidence-Based Toxicology Collaboration, Johns Hopkins University Bloomberg School of Public Health, Baltimore, MD USA; 6 Lancaster Environment Centre, Lancaster University, Lancaster, UK; 7 Seh Consulting + Services, Paderborn, Germany

**Keywords:** Causality analysis, Evidence-based toxicology, Mechanistic inference, Network toxicology, Multi-omics integration, Adverse outcome pathway, Exposure–disease linkage, Systems biology, Risk assessment

## Abstract

This paper examines why robust causality assessment is central to toxicology yet increasingly difficult in networked biological systems, including Adverse Outcome Pathway (AOP) networks and multi-omics datasets. Classical causal frameworks (e.g., Koch/Dale reasoning and Bradford Hill-type considerations) remain useful for scoping, but they offer limited operational guidance for multifactorial, nonlinear, and feedback-dominated mechanisms. We therefore synthesize a modern toolbox for causality analysis in toxicology spanning directed acyclic graphs for explicit causal assumptions, quantitative and probabilistic approaches for integrating evidence across key events, and network/*dynamical* methods that help identify influential hubs and points of vulnerability in AOP networks. Building on evidence-based toxicology, we propose an operational workflow that links (i) structured scoping and model specification, (ii) protocolized evidence retrieval across in vivo, in vitro, in silico and human data streams, (iii) risk-of-bias appraisal and quantitative synthesis of effect sizes and dose–response, (iv) mechanistic integration and targeted perturbation assays, and (v) translation via dual-strand certainty rating (mechanistic vs difference-making evidence) and Evidence-to-Decision tables. We discuss how explainable AI can support scalable integration and transparency. A worked developmental neurotoxicity example illustrates how this pipeline can support regulatory recommendations while explicitly documenting uncertainty.

## Introduction

Causal relationships are of critical importance in toxicology. They allow to move beyond correlations, which even may be spurious, and build a mechanistic foundation for safety assessments. While correlation can suggest connections between variables, it does not imply causation. Strong, consistent, repeatable, dose-dependent, coherent, temporal and predictive relationships need to be shown experimentally to demonstrate that a change in stimulus determines a directional change in effect.

**Fig. 1:**
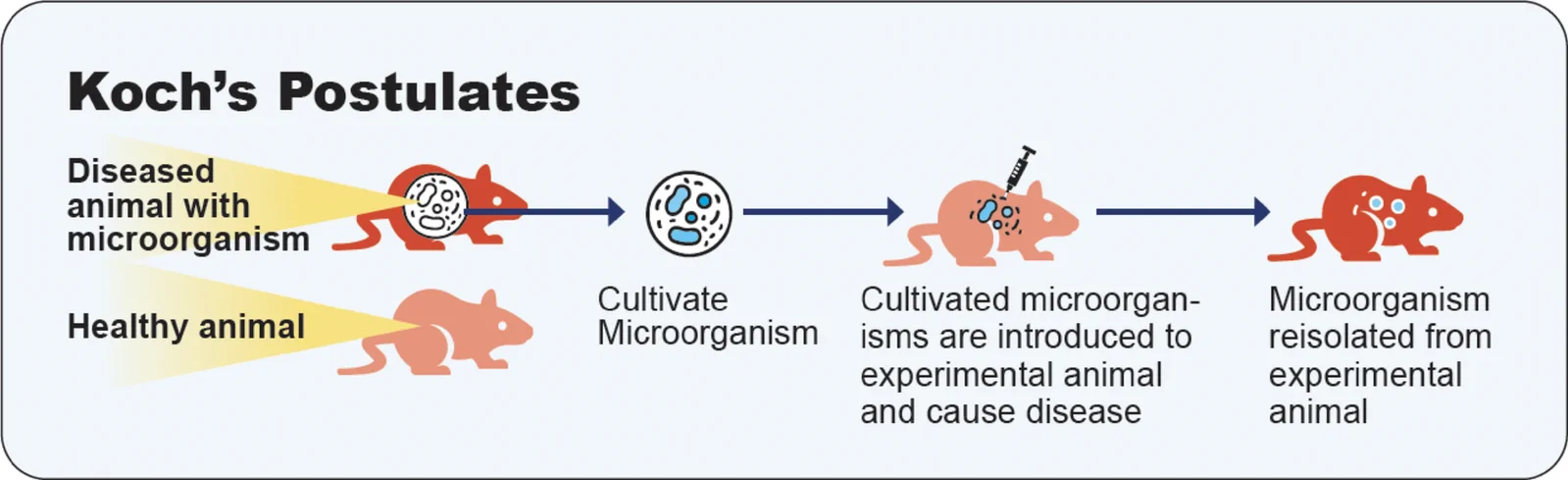
Schematic presentation of the Koch’s Postulates

**Fig. 2:**
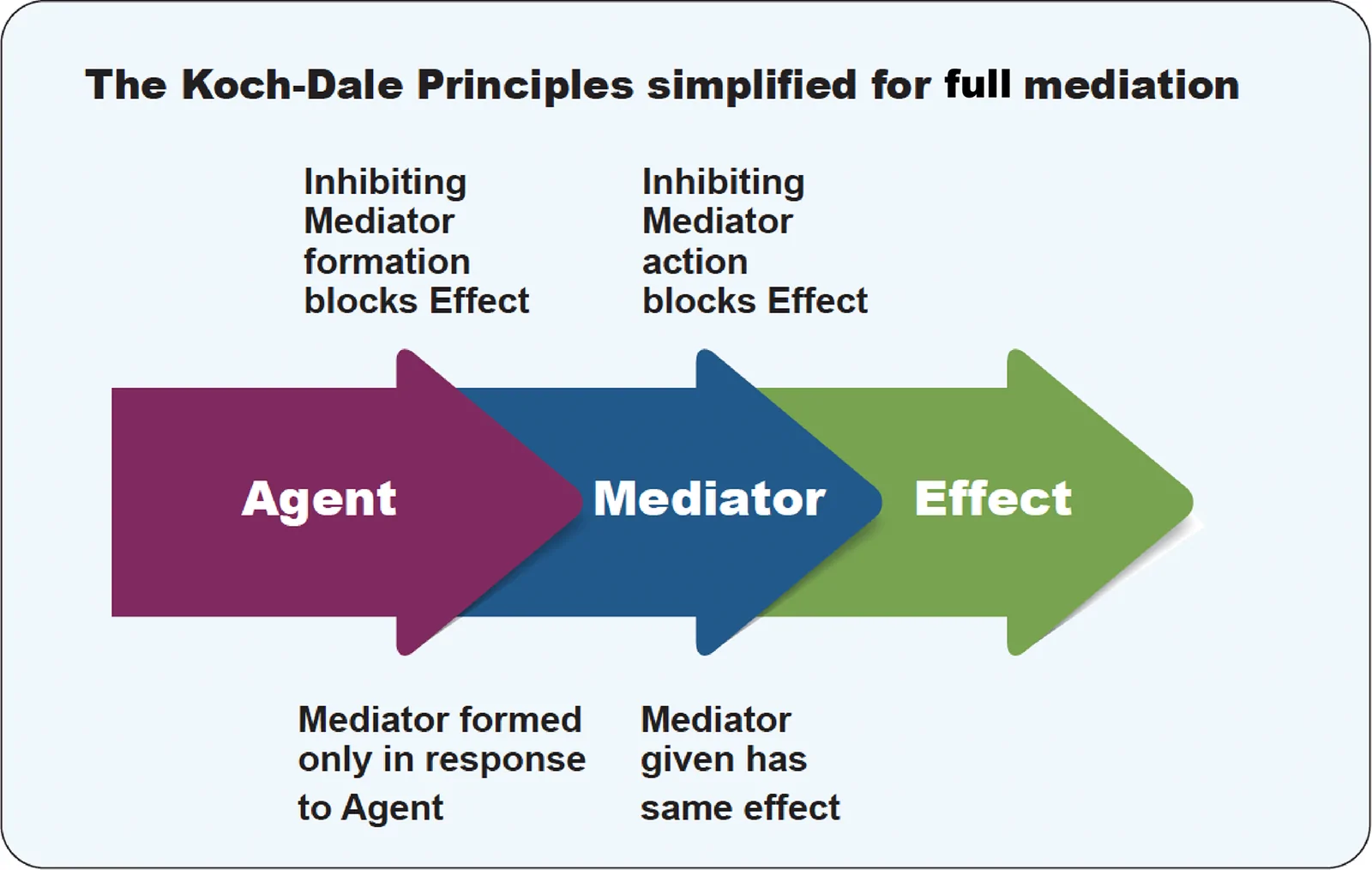
Dale’s adaptation of the Koch principles to general biological mediators

The early work of Robert Koch on infectious diseases in the late 1800s provided the first experimental framework for causation, the Koch’s Postulates, which describe what is required for the identification of the responsible pathogen, i.e., isolating the microorganism, growing it in pure culture, reproducing the disease in a healthy organism, and re-isolating the microorganism. The core concept of Koch’s Postulates, illustrated in Fig. 1, is that a mediator is present in disease states but absent in health, that giving the mediator elicits the disease, and blocking the mediator prevents the disease. After Dale’s adaptation, this concept was further developed into the “Koch-Dale principles” (Fig. 2).

This was later adapted to other fields like physiology by showing a mediator is present in disease states but absent in health, that giving the mediator elicits the disease, and blocking the mediator prevents the disease. The Koch-Dale criteria, named after Robert Koch and Henry Dale (Dale 1929), provide a framework for demonstrating that a specific mediator causes a biological effect (Fig. 2). Building on Koch’s postulates for infectious agents, Dale proposed in the 1920s that a mediator should be present in disease states but not healthy states, that administering the mediator should reproduce the effect, and that blocking the mediator should prevent the effect.

**Fig. 3:**
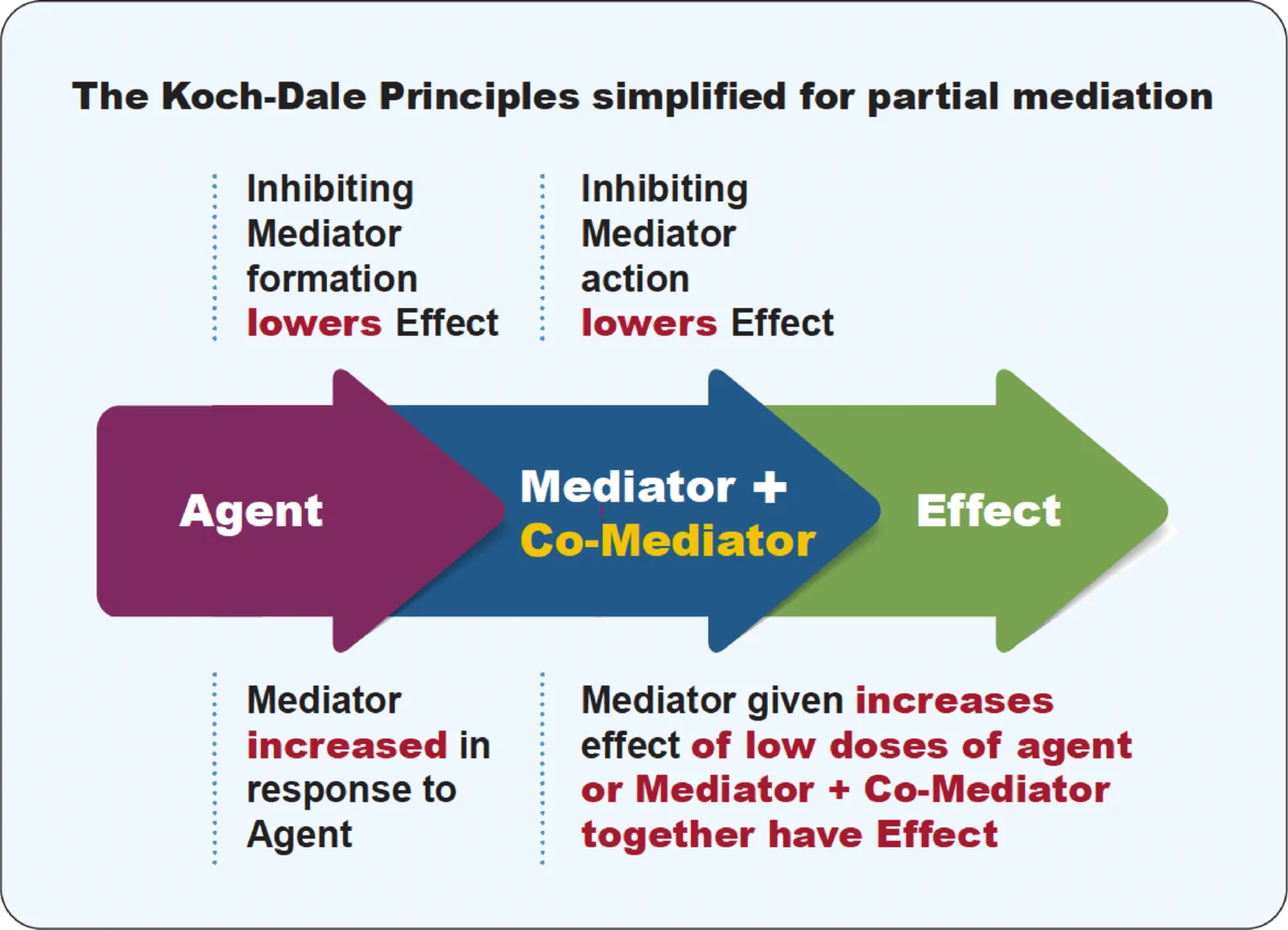
The Koch-Dale Principles adapted for mediation of effect by more than one mediator

This set of logic is broadly applicable for determining if any agent (e.g., a gene, metabolite, toxicant) acts through a particular mediator to produce an effect. Critically, the criteria require not just showing a correlation between the mediator and effect but demonstrating that experimentally manipulating the mediator in both directions (mimicking and inhibiting its activity) consistently impacts the effect in question. While the directness of this approach is compelling, the mediator may not always be the sole cause, but rather a key component of a sufficient causal mechanism, requiring adaptation (Fig. 3). Such partial mediation is for example seen where several cytokines released together have redundant activities, so blocking one at best lowers effects but does not blunt them. Nevertheless, the Koch-Dale criteria remain a cornerstone for assessing causality in biological systems.

**Fig. 4:**
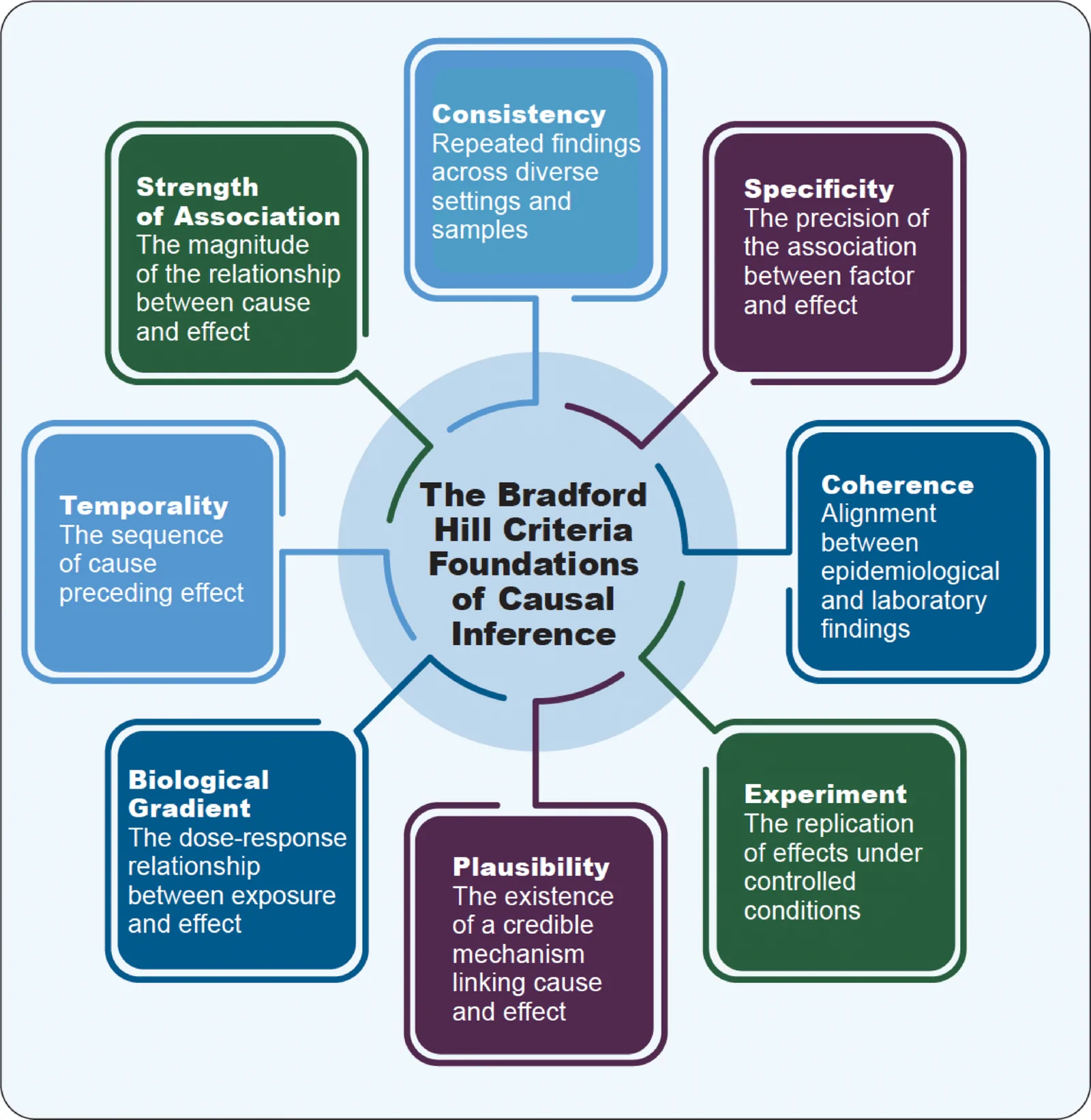
Simplified representation of the Bradford-Hill criteria for causation

In the 1960s, Sir Bradford Hill proposed (Hill 1965) a set of criteria to help establish likely causal relationships from epidemiological data in the absence of clear experiments (Fig. 4). These criteria, which included the strength, consistency, specificity, temporality, biological gradient, plausibility, coherence, and analogy of the associations, became a key checklist for inferring causality. While meeting the criteria does not guarantee causality, it provides an important framework for assessing the weight of evidence.

Over the past decade, toxicology has increasingly embraced mode of action and adverse outcome pathway approaches to organize mechanistic data and develop predictive models. Cataloging and validating pathways of toxicity (PoT) (Kleensang et al. 2014), the molecular mechanisms by which chemicals perturb biological systems, was the aim of the Human Toxome project (Bouhilfd et al., 2015). Demonstrating that a test system reliably captures key events in an already established pathway provides a basis for mechanistic validation (Hartung et al. 2013).

However, given the complexities of biology, toxicology will need to evolve to model perturbations of dynamic, interacting networks. New approaches in systems toxicology, integrating large-scale omics data, literature mining, and computer modeling, hold promise for simulating the effects of substances on cells, tissues, and even virtual organs and patients. While many challenges remain, moving from empirical animal testing to mechanistic, human-relevant in vitro and in silico models is essential for modernizing risk assessments.

## Bradford hill criteria

The Bradford Hill criteria, proposed by Sir Austin Bradford Hill in 1965, provide a framework for evaluating the strength of evidence for a causal relationship between an exposure and a health effect, particularly when direct experimental evidence is lacking (Fig. 4). These criteria have been widely used in epidemiology and public health to assess the likelihood that an observed association is causal. The Bradford Hill criteria consist of nine aspects to consider when assessing causality. They are fundamental to the GRADE approach (Schünemann et al. 2011) as we will see in chapter 3.

**Fig. 5:**
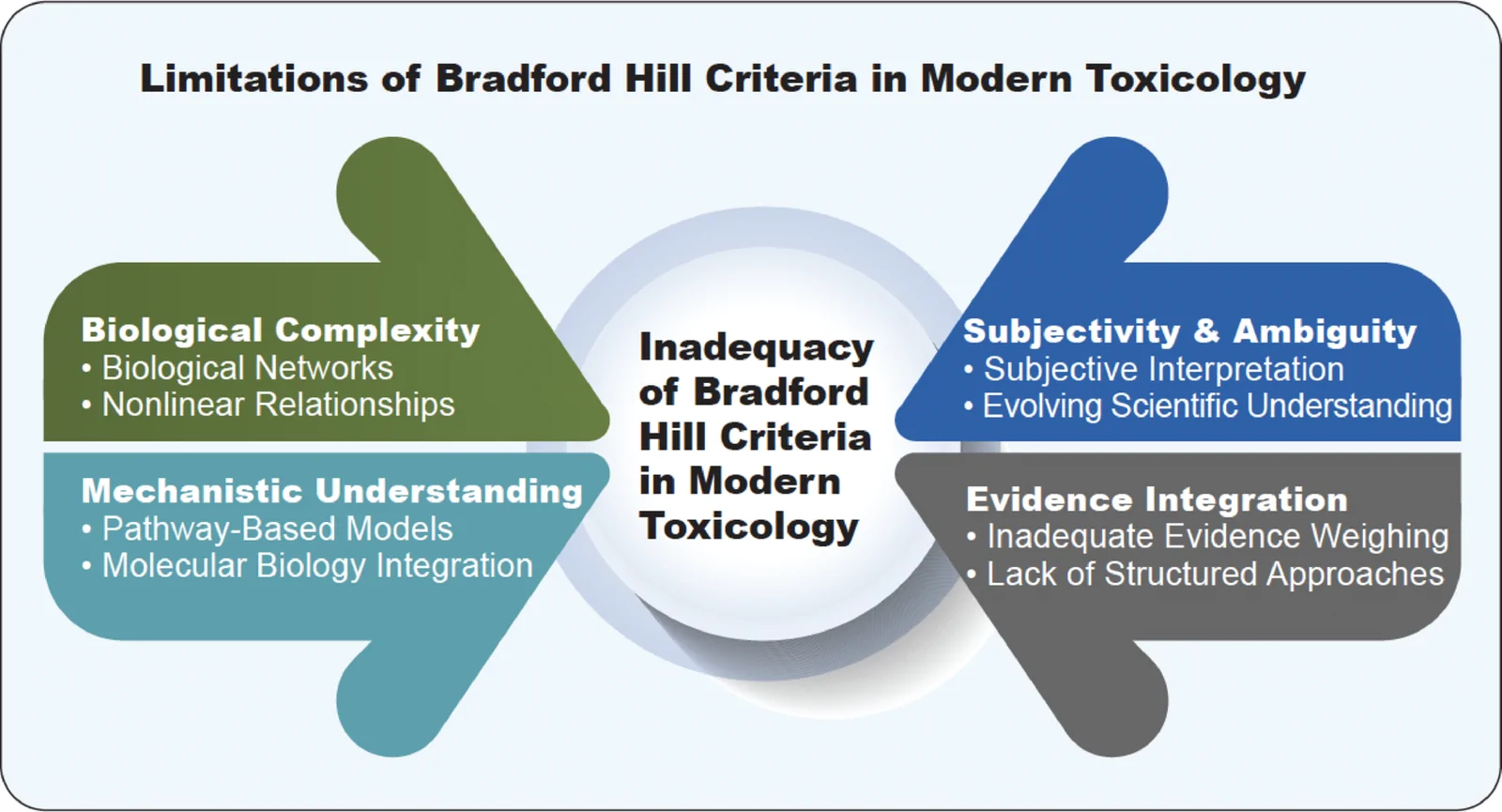
Limitations of the Bradford-Hill criteria for modern toxicology

While these criteria provide a useful guide, they were developed primarily with linear, single-agent causal relationships in mind. However, most biological systems, from signaling pathways within cells to organ systems and ecological networks, are complex webs of interacting components. In these networked systems, causation may be multifactorial, involving the interplay of multiple agents and pathways. Effects may propagate through the network in non-linear ways, with feedback loops, redundancies, and emergent properties (Fig. 5).

Evans’ postulates (1976) offered a more formalized and testable model. In the context of Evidence-based Toxicology (EBT), where emphasis is increasingly placed on mechanistic understanding, transparency, and reproducibility, it is essential to assess the utility and limitations of both approaches. Here, we critically compare the two frameworks and evaluates their relevance for modern toxicology, particularly as the field moves toward systems toxicology and pathway-based approaches.

The strength of Hill’s approach lies in its flexibility and breadth, serving as a heuristic rather than a checklist. However, several of the criteria are subjective (e.g., plausibility, coherence), redundant (e.g., strength and gradient), as they have not been sufficiently operationalized with the notable exception of the OECD Adverse Outcome Pathway (AOP) approach, or difficult to apply in the context of non-linear, multifactorial, or mechanistically complex biological systems (Hall 2023). Importantly, the traditional Bradford Hill framework does not prescribe quantitative thresholds when a criterion is fulfilled, lacks structured evidence integration methods, and does not explicitly require mechanistic validation. In essence, causality is a tough challenge, even at the macro level (exposure-outcome associations). In toxicology, we are very concerned with establishing causality through understanding of biological mechanisms, while the macro-level considerations do not directly apply to mechanistic modelling.

**Fig. 6:**
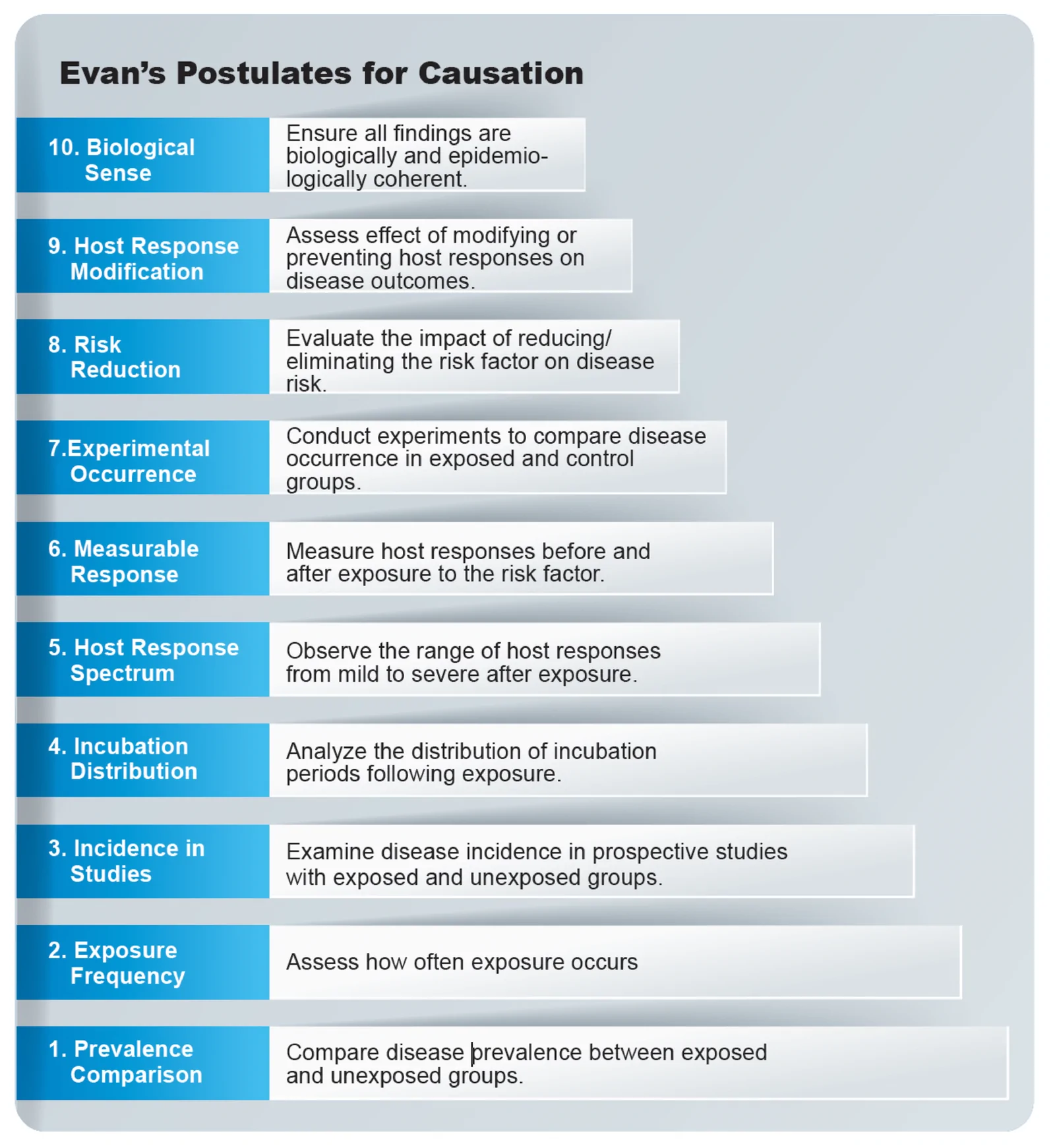
Evans’ Postulates for Causation. Evans (1976) developed a set of 10 postulates applying causation logic

In 1976, Alfred S. Evans introduced a set of postulates intended to apply causation logic beyond infectious diseases (Fig. 6).

Evans’ postulates aim to integrate quantitative rigor, experimental reproducibility, and biological coherence, making them well-suited for structured evaluation such as those used in systematic reviews or EBT frameworks.

- *Quantitative Rigor*: Evans’ postulates provide clearer operational criteria for causal inference, such as requiring measurable differences in prevalence and incidence, or evaluating dose–response gradients. Bradford Hill, in contrast, offers qualitative guidance that lacks threshold-based interpretation.
- *Mechanistic Integration*: While Hill includes plausibility, biological gradient and coherence, Evans directly calls for measurable host responses, aligning better with mechanistic frameworks such as Adverse Outcome Pathways (AOPs).
- *Temporal and Experimental Requirements*: Both frameworks emphasize temporality and experimental support. However, Evans is more specific in demanding experimental replication and reversibility, which align with validation practices in modern toxicology.
- *Relevance to EBT and Systems Toxicology*: Mechanistic validation, a core tenet of modern EBT, emphasizes causality through pathway-level resolution, reproducibility, and predictive validity. Evans’ approach, with its insistence on reproducibility and host response, is more compatible with mechanistic validation schemes than Bradford Hill’s narrative-oriented framework.

Bradford Hill criteria remain valuable for hypothesis generation and historical interpretation, but they do not accommodate the integration of multi-omics data, in vitro and in silico models, or bioinformatics tools as readily as Evans’ postulates do.

Several other modifications and extensions to the original Bradford Hill criteria have been proposed over the years to address specific contexts or to incorporate new scientific understanding. Some key examples include:

1. *Rothman’s Sufficient-Component Cause Model*: Kenneth Rothman (1976) proposed that a cause should be viewed as a sufficient component within a causal mechanism, rather than a necessary one. This allows for multifactorial causation and the idea that a specific cause may not always be required if other sufficient components are present.
2. *Susser’s Criteria*: Mervyn Susser (1991) suggested additional criteria, such as the predictive performance of a causal hypothesis and its ability to explain existing data. He also emphasized the importance of considering alternative explanations.
3. *Bunge’s Criteria*: Mario Bunge (1979), original work published 1959, proposed a set of criteria grounded in a philosophical understanding of causation, including the notion that causes must precede effects, that causal relationships should be invariant under a range of conditions, and that causal explanations should be consistent with established scientific theories.
4. *Russo-Williamson Thesis*: Federica Russo and Jon Williamson (2007) argued that both mechanistic and difference-making evidence are required to establish causal claims in the health sciences. Mechanistic evidence sheds light on the biological plausibility of a causal relationship, while difference-making evidence (e.g., from randomized controlled trials) demonstrates the effect of manipulating the cause.
5. *Howick’s Modifications*: Jeremy Howick and colleagues (2009) suggested revisions to the Bradford Hill criteria to improve their applicability and avoid common misinterpretations. For example, they argue that temporality should be considered a necessary condition for causality, while coherence, plausibility, and analogy should be viewed as one criterion related to existing theory.
6. *WHO/IPCS Mode of Action framework*: Bette Meek and colleagues (2014) developed the WHO/IPCS Mode of Action framework explicitly “*evolved*” the original Bradford Hill criteria to better assess the weight of evidence for mechanistic hypotheses rather than epidemiological associations. This adapted framework prioritizes “*biological concordance*” and the “*essentiality of key events*,” thereby shifting the analytical focus toward experimental verification of causal linkages within a pathway. By structuring the analysis around these evolved considerations, the framework significantly enhances transparency and consistency when determining the human relevance of toxicological data
7. *Network Approaches*: With the growing understanding of biological systems as complex networks, some researchers have proposed modifications to account for multifactorial causation, feedback loops, and non-linear dynamics (Barabási et al. 2011; Fedak et al. 2015). This includes considering the strength and consistency of associations in the context of network states and using experimental perturbations and computational models to map causal pathways.

Applying the Bradford Hill criteria to networked systems requires a more nuanced approach. In these complex environments, the strength and consistency of associations are often obscured because the effect of any single component is modulated by the overall state of the network. Specificity is frequently lost as perturbations “*ripple*” through the system in unpredictable ways, and dose–response relationships often defy linearity due to tipping points and buffering effects. Consequently, biological plausibility and coherence must now be assessed within the context of these dynamic network interactions rather than as static properties.

Despite these challenges, the core principles of Bradford Hill remain essential when adapted. Temporal relationships, where a change in one network node consistently precedes an effect in another, remain our best tool for mapping causal pathways. Similarly, experimental evidence retains its primacy, though now achieved through sophisticated manipulations like gene knockouts or pharmacological interventions to probe specific components. Even analogy finds new relevance, where similarities between network perturbations can suggest common causal mechanisms.

To operationalize this, we must integrate these classic principles with modern tools: omics technologies, network analysis, and multiscale modeling allow us to characterize these complex responses in ways traditional observation cannot. By combining Hill’s foundational heuristics with the quantitative rigor of Evans’ postulates, which better support the structured synthesis of heterogeneous evidence required by EBT, we can build a framework capable of handling multifactorial causation. In Chapter 10, we attempt to define this hybrid framework, demonstrating how to merge mechanistically anchored data with operational causal inference to support regulatory decision-making.

## The evidence-based toxicology movement and its reliance on causation

**Fig. 7:**
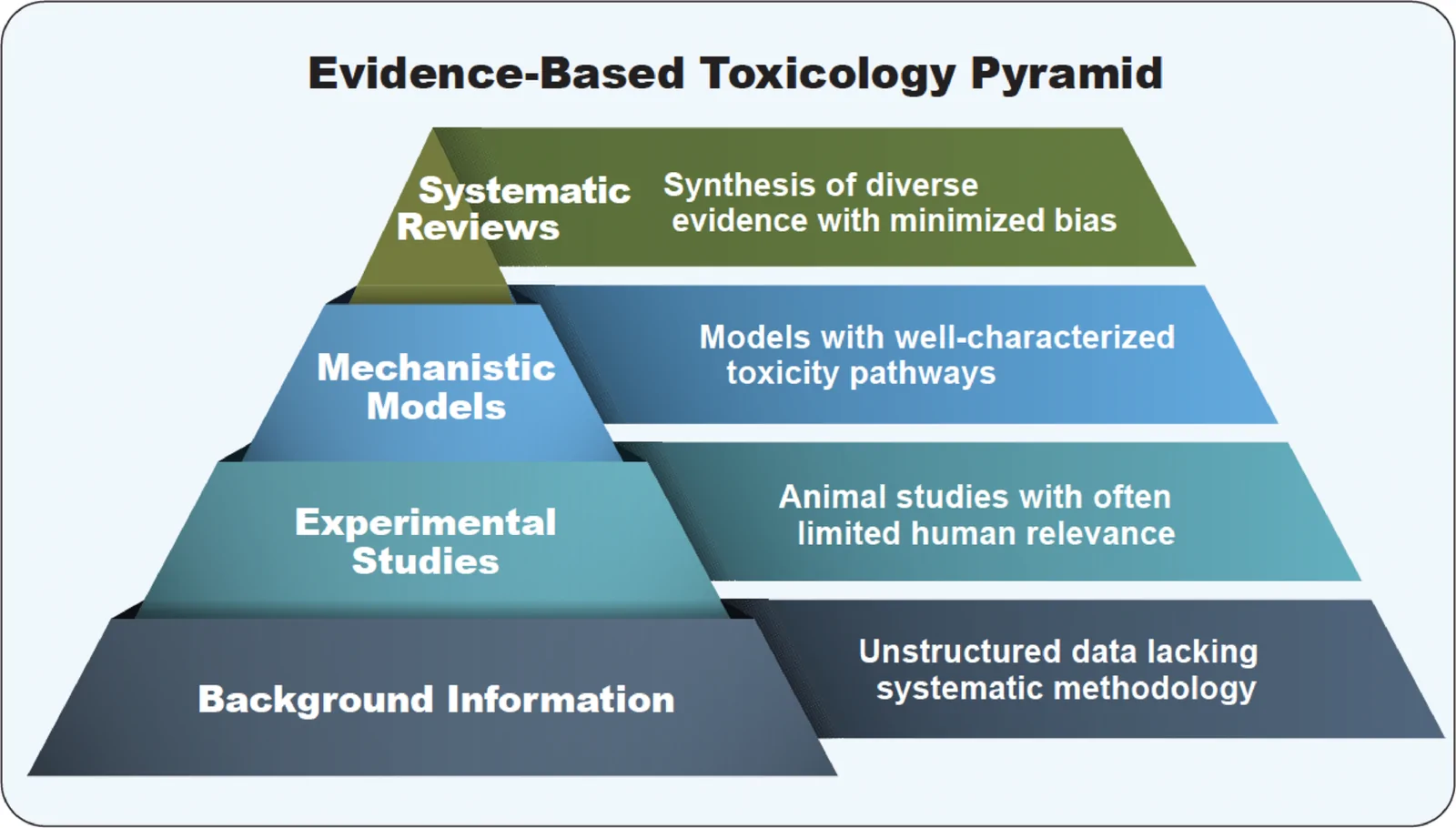
The evidence pyramid of toxicology. Adapted here to toxicology, the evidence pyramid shows that most information in toxicology is unstructured. Intervention studies in animals are black-box type of experiments as causality is mostly not known or addressed. Mechanistic models build on (verified) pathway hypotheses. Only few systematic assessments integrate available evidence in an objective and transparent way

Evidence-based toxicology (EBT) (Hoffmann and Hartung 2006; Hartung 2009; Hartung and Tsaioun 2024) calls for structured, transparent, and systematic methodology adapted from evidence-based medicine (EBM), which optimally inform causation assessment (Guzelian et al. 2005) (Fig. 7).

Rather than relying on authority or expert consensus alone, EBT emphasizes the critical appraisal of all available evidence using predefined criteria. It distinguishes between nomological possibilities (i.e., theoretical risks) and epistemic possibilities (i.e., known causal relationships) and only the latter are considered genuine risks. The EBT framework as suggested by Guzelian et al. (2005)) consists of three sequential steps: (1) Collecting and evaluating relevant data (e.g., source, exposure, dose, diagnosis), (2) Collecting and evaluating relevant knowledge through systematic review (Hoffmann et al. 2017) of the literature, and (3) Synthesizing data and knowledge to determine causation based on explicit criteria (general causation, dose–response, temporality, alternative causes, and coherence). Noteworthy, EBT, as understood now, is a set of principles, not a set of specific methodological approaches. Causation in EBT is inferred when evidence satisfies multiple criteria, including strength, consistency, specificity, temporality, biological gradient, plausibility, coherence, experimental evidence, and predictive performance. We have to be careful, though, not to conflate protocols with issues of certainty of evidence. These criteria are drawn from and refined versions of the Bradford-Hill criteria, but EBT emphasizes that no single criterion is sufficient, and all must be interpreted in the context of the evidence hierarchy and mechanistic understanding. Importantly, EBT stresses that evidence must be evaluated using a transparent protocol—often involving meta-analyses, structured checklists, and quality grading of individual studies—to avoid bias and improve reproducibility. This serves against untested assumptions with respect to causal claims and supports more scientifically grounded decision-making in toxicology and risk assessment.

Moving from evidence integration to resulting decisions, the GRADE (Grading of Recommendations, Assessment, Development and Evaluation)^1^ and Evidence-to-Decision (EtD) frameworks represent systematic, transparent methodologies for assessing the certainty of research evidence and translating it into informed healthcare recommendations or policy decisions. The GRADE Approach is a structured framework for assessing certainty of research evidence and using evidence to support decision-making. GRADE consists of two elements: a Certainty of Evidence (CoE) assessment for evaluating and describing certainty in the evidence for effects on health of an intervention or exposure; and an EtD framework for selecting a key set of prespecified but flexible criteria when developing recommendations about health interventions, health policy or coverage decisions, or occupational and environmental health policy, and for describing how strongly the recommendation is being made.

The CoE framework (Whaley et al. 2022) is a modernization and update of the Bradford Hill criteria. It rates certainty that an observed effect exceeds a specified threshold (e.g., is an absolute benefit of reducing an unwanted outcome of 5 cases per 1000), or is within a specified range (e.g., is a moderate harm, with moderate defined as increasing the incidence of an unwanted outcome between 5 and 20 cases per million).

The CoE framework works by assuming evidence is of high certainty, then rating down for limitations in the evidence that reduce certainty that the effect exceeds the threshold or is within the range. The downgrade factors include limitations in study design and execution (that introduce risk of bias into a study), inconsistency (unexplained differences in results of otherwise similar studies), and indirectness (differences between the features and context of the evidence, and the features and context of the situation to which the evidence is being applied), among others.

The GRADE EtD framework supports decision-making by encouraging, in addition to certainty about the harms and benefits of an exposure or intervention, systematic consideration of a range of additional factors that are critical for optimally choosing between the possible responses to the situation in which potential harm or benefit is occurring. These factors include but are not limited to the balance of desirable and undesirable effects of the proposed responses, the cost-effectiveness of the responses, the acceptability of the responses to those impacted by them, and the impact of the responses on social equity.

Together, these evidence-based synthesis and decision-support methods ensure that clinical guidelines, coverage decisions, and health policies are grounded in rigorous evaluation of available evidence while explicitly considering the broader contextual factors that influence real-world implementation and outcomes, making them particularly valuable in evidence-based toxicology where complex causal relationships and heterogeneous evidence sources require systematic integration for regulatory decision-making. Notably, one of four working groups of the EBT collaboration^2^ is currently translating EtD to toxicology (Senerth et al. 2025).

In conclusion, demonstrating causation, not simply correlation, is fundamental to building scientific confidence in new predictive models. Evidence-based approaches, like systematic reviews, can help establish mechanistic validity. Notably, the advent of AI supporting systematic reviews has strongly increased their use (Bozada et al. 2021; Kleinstreuer and Hartung 2024; Schmidt et al. 2025). Though our understanding may never be perfect, we must use the best available science to make decisions and refine our knowledge of causal pathways to better protect human health.

## Causality analysis in networked systems

Understanding causality in biological systems presents a fundamental challenge: while traditional frameworks like the Bradford Hill criteria provide essential heuristics, they were not optimized for the complex, non-linear networks that define biology. Biological systems operate through webs of interacting components, such as gene regulatory and signaling networks, where perturbations propagate non-linearly, generating emergent properties and feedback loops that defy simple linear causal assumptions.

### Adverse outcome pathways: a framework for causality

In toxicology, the Adverse Outcome Pathway (AOP) framework (Leist et al. 2017; de Vries et al. 2021) structures these causal chains from a Molecular Initiating Event (MIE) to an Adverse Outcome (AO). AOPs facilitate the integration of mechanistic data from in vitro and in silico models to support risk assessment. However, while early AOPs often represented simplified, direct relationships, “*networked AOPs*” are now emerging to better capture biological complexity. They also facilitate the use of mechanistic data from in vitro and in silico models to support causal inference and risk assessment (Hoffmann et al. 2022). Crucially, the confidence in any AOP, thus, the certainty associated with the causal chain, depends heavily on the certainty of the evidence used to construct it.

### Challenges and approaches to networked causality

**Fig. 8:**
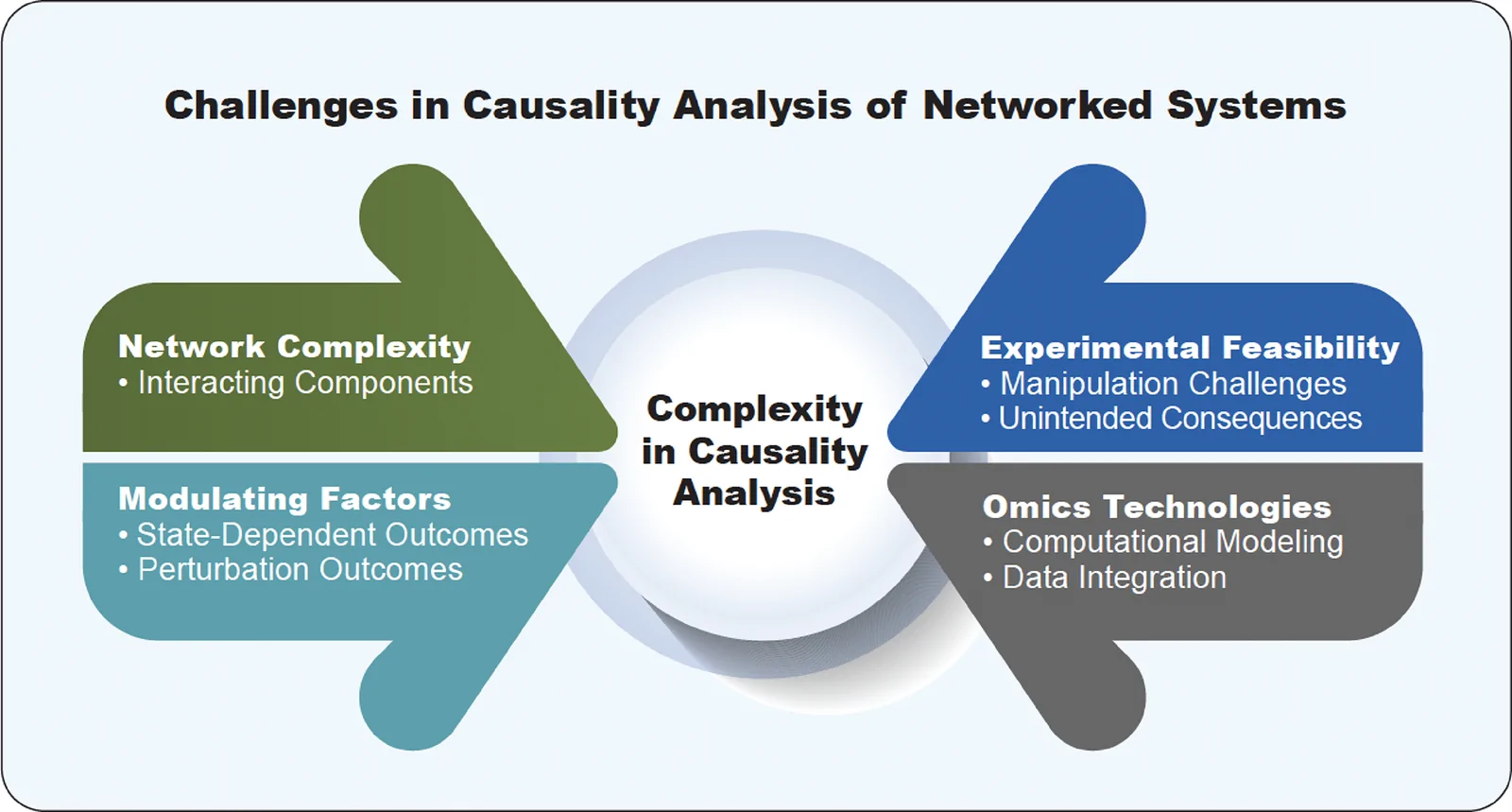
Challenges in causality analysis of networked systems

Analyzing causality in these systems requires a methodological shift. The sheer complexity of interacting components and modulating factors makes isolating specific cause-effect relationships via traditional experimental manipulation difficult (Fig. 8). To address this, toxicology must integrate high-content multi-omics technologies with computational network analysis (e.g., graph theory, dynamic modeling) to identify key nodes and predict the effects of perturbations. This integration supports a transition from viewing causes as deterministic (“*necessary and sufficient*”) to understanding them as probabilistic (Maertens et al. 2022, 2024, 2025) and context-dependent. In this view, causality is defined not by linear links, but by the strength and consistency of associations across multiple lines of evidence within the network state (Hartung et al. 2012).

### Conclusion

Causality analysis in networked biological systems is a challenging but essential task for understanding the impacts of toxicants and other stressors on living systems. The AOP framework provides a structured approach for organizing evidence of causal relationships across levels of biological organization. However, fully elucidating the multifactorial and non-linear causal pathways within complex networks will require the integration of computational modeling, multi-omics data, and high-throughput experimentation.

By embracing a systems perspective and leveraging new tools and approaches, we can work towards a more robust and predictive understanding of causality in biological networks. This understanding will be critical for advancing the fields of toxicology, risk assessment, and precision medicine, enabling the development of safer chemicals, more effective therapies, and personalized interventions to promote health and prevent disease.

## Cellular resilience and network causality

### Resilience as a causal modifier

The concept of cellular resilience (Smirnova et al. 2015) challenges linear toxicology by introducing non-linear dynamics where outcomes are determined by the balance between damage and adaptive responses to earlier stressors. Unlike simple causal chains, cells utilize complex networks to buffer against perturbations, redirect metabolic resources, and modify gene expression to establish new homeostatic states (Krug et al. 2014; Harris et al. 2018). Resilience mechanisms fundamentally alter causal relationships through threshold effects preventing adverse outcomes until defensive capacities are overwhelmed, and through redundancy, where compensatory pathways can mask direct causal links. Additionally, prior exposures can modify future responses via hormesis or cellular memory, meaning simple linear models often fail to capture the context-dependent nature of toxicity.

### Temporal dynamics and experimental implications

Causality in resilient systems must be analyzed longitudinally across three distinct phases: the immediate stress response, a recovery phase where cells return to homeostasis or establish a new steady state, and long-term adaptation involving epigenetic modifications. The temporal dimension involves long-term adaptation, which may include epigenetic modifications (Hartung et al. 2025b) that alter gene expression patterns, changes in cellular sensitivity to various stimuli, and the establishment of cellular memory effects that influence responses to future exposures. These temporal dynamics challenge simple causal models and suggest the need for longitudinal approaches to causality assessment. Consequently, experimental designs must evolve to include recovery periods and assessment of repeated exposures to capture reversibility and adaptive trajectories. Analytical frameworks must identify “*tipping points*” (Shah et al. 2016; Saili et al. 2020), which represent critical thresholds beyond which resilience mechanisms fail and adverse outcomes become irreversible. Network compensation provides yet another layer of complexity, with redundant pathways potentially masking causal relationships and alternative mechanisms achieving similar functional outcomes through different means. Understanding these modifying effects is crucial for accurate causal analysis in toxicology. The presence of such complex modifiers means that simple linear causal relationships may fail to capture the true nature of toxicological responses.

### Systems-level assessment

To address these complexities, causality analysis requires a “*systems-level assessment*”, aka Systems Toxicology (Hartung et al. 2017), that integrates multiple endpoints to capture emergent properties, i.e., characteristics of the whole system that cannot be predicted from individual components alone. This approach moves beyond identifying necessary causes to evaluating network stability and robustness in the face of perturbation. Future research must focus on developing tools capable of mapping these resilience pathways and compensatory mechanisms to better predict how cells modify their behavior in response to toxic challenges. To address the complexity of causal relationships in toxicology, the “3S” framework (Smirnova et al. 2018) integrates three complementary methodologies: Systematic, Systemic, and Systems Biology/Toxicology. The framework’s value lies in its cyclical integration: systematic reviews guide model development, systemic models generate data, and systems biology refines predictions. Implementation requires addressing challenges in quality assurance, data standardization, and regulatory validation. Future developments will focus on seamless data flow and increasingly sophisticated organ-on-chip systems to enhance predictive power for human health outcomes.

### Implications for causality assessment

The integration of resilience concepts into causality analysis has several practical implications for toxicological research and assessment. Experimental design must now incorporate temporal profiling and include appropriate recovery periods. Whether we like it or not, animal experimentation has an edge here, which microphysiological systems only start to catch up with (Hartung and Smirnova 2025; Marx et al. 2016, 2020, 2025). These models allow researchers to study inter-organ communication and recovery processes as well as to study systemic interactions and whole-organism responses advancing drug development (Roth et al. 2021) and investigative toxicology (Beilmann et al. 2019). Studies should consider the effects of repeated exposures and assess adaptive responses that may develop over time. Data analysis approaches need to integrate network perspectives and consider system-level properties rather than focusing solely on individual pathways. The analysis of temporal dynamics becomes crucial, as does the assessment of reversibility in observed effects. Interpretation of results must recognize the context-dependency of responses and consider cellular history as a crucial factor. Attention must be paid to adaptive capabilities, and assessment of long-term consequences becomes essential for understanding the full impact of toxic exposures.

The integration of resilience concepts into causality analysis suggests several important directions for future research. (1) In terms of methodological development, we need new tools capable of analyzing complex biological networks in their entirety, especially improved temporal profiling methods to track cellular responses over extended periods and the respective enhanced data integration approaches to make sense of the vast amounts of information. (2) Conceptual advancement to refined models of biological causality that can account for the complex interplay between damage and resilience mechanisms is critical to understand how cells modify their behavior to future toxic challenges.

Understanding cellular resilience fundamentally transforms our approach to causality in toxicology. Moving beyond simple cause-and-effect relationships, we must consider complex networks of response, adaptation, and recovery. The future lies in the continued integration of network approaches, temporal analysis, and systems-level thinking. The intersection of resilience and causality represents a frontier in toxicological research, one that promises to revolutionize our understanding of how cells respond to and recover from toxic challenges.

## Exploring causation in complex networks: key concepts and methods

In the realm of complex systems, understanding causation is a crucial yet challenging task. Complex networks, which are ubiquitous in nature and society, exhibit intricate patterns of interactions and dependencies that can obscure the true causal relationships between variables. This chapter delves into the key concepts and methods used to unravel causation in complex networks, providing a comprehensive overview of the tools and techniques employed beyond toxicology.

### Key tools to identify causation in complex networks

**Fig. 9:**
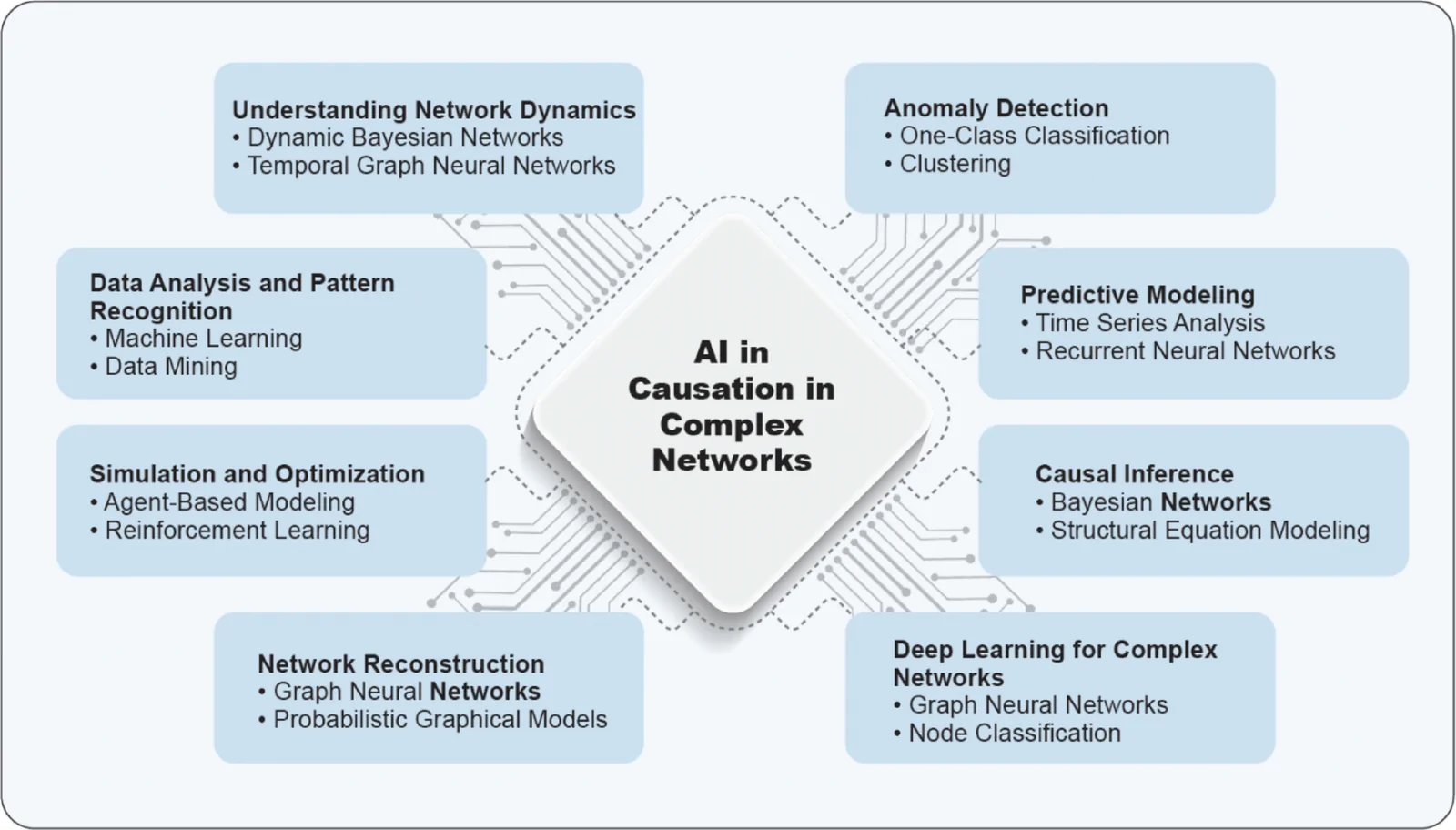
Tools for identifying causation in complex networks

Figure 9 shows the most important tools to explore causality.

#### Causal graphs: visualizing causality

Causal graphs (Koller and Friedman 2009) serve as a fundamental tool for representing and analyzing causal relationships in complex networks. In a causal graph, nodes represent variables, and directed edges signify the causal influence of one variable on another. These graphical models provide an intuitive and visually appealing way to depict the intricate web of cause-and-effect relationships within a network. By constructing causal graphs, we can gain insights into the structure and dynamics of complex systems. These graphs enable the identification of direct and indirect causal paths, feedback loops, and potential confounding factors. Moreover, causal graphs facilitate the formulation of testable hypotheses and guide the design of experiments or interventions to validate causal claims.

#### Causal inference: unraveling cause and effect

Causal inference (Pearl 2009) lies at the heart of understanding causation in complex networks. It involves the process of determining whether a change in one variable can be attributed to a change in another variable. In other words, causal inference seeks to establish the presence and direction of causal relationships between variables. Various statistical methods are employed for causal inference, including regression analysis and Bayesian inference. Regression analysis examines the relationship between a dependent variable and one or more independent variables, aiming to identify the strength and significance of the causal effect. Bayesian inference, on the other hand, incorporates prior knowledge and updates beliefs based on observed data, providing a probabilistic framework for causal reasoning.

#### Granger causality: predictive power as a measure of causality

Granger causality, introduced by Nobel laureate Clive Granger (Shojaie and Fox 2022), is a statistical concept that assesses causality based on the predictive power of variables. According to Granger causality, if a signal X “Granger-causes” a signal Y, then the past values of X should contain information that improves the prediction of Y beyond what can be predicted solely based on the past values of Y. Granger causality tests are widely used in time series analysis to determine the direction of information flow and identify causal relationships. By comparing the predictive power of different variables, one can infer the presence and strength of causal influences within a network. However, it is important to note that Granger causality is based on temporal precedence and does not necessarily imply true causation in the philosophical sense.

#### Transfer entropy: quantifying information flow

Transfer entropy, proposed by Thomas Schreiber (2000), is a non-parametric statistic that measures the directed transfer of information between random processes. It quantifies the amount of information that one variable provides about the future states of another variable, beyond what is already contained in the past states of the latter variable. Transfer entropy is particularly useful for analyzing complex networks with nonlinear interactions and detecting asymmetric information flow. By calculating transfer entropy between pairs of variables, researchers can identify the direction and strength of causal influences, even in the presence of noise and confounding factors. This method has found applications in various domains, including neuroscience, economics, and climate science.

#### Complex network analysis: unveiling structural patterns

Complex network analysis (Strogatz 2001) offers a powerful framework for studying the structural properties and dynamics of complex systems. By representing a system as a network of nodes and edges, researchers can apply a wide range of network science methods to uncover hidden patterns and insights. Community detection algorithms, such as modularity maximization and stochastic block models, are used to identify densely connected groups of nodes that may share similar characteristics or functions. Centrality measures, including degree centrality, betweenness centrality, and eigenvector centrality, help identify the most influential or critical nodes in a network. These structural features can provide valuable clues about the underlying causal mechanisms and the flow of information or influence within the network.

#### Dynamical systems theory: capturing network dynamics

Dynamical systems theory (Strogatz 2015) offers a mathematical framework for describing and analyzing the behavior of complex systems over time. It provides tools for studying the evolution of network states, the emergence of collective behavior, and the stability and resilience of complex networks. By formulating a network as a dynamical system, researchers can investigate how the network’s structure influences its dynamics and vice versa. Concepts such as attractors, bifurcations, and phase transitions can shed light on the long-term behavior and critical points of the system. Dynamical systems theory also enables the study of feedback loops, self-organization, and the propagation of perturbations through the network.

#### Conclusion

Understanding causation in complex networks is a multifaceted endeavor that requires a combination of conceptual frameworks, statistical methods, and computational tools. Causal graphs provide a visual representation of causal relationships, while causal inference techniques enable the quantification and testing of causal claims. Granger causality and transfer entropy offer measures of predictive power and information flow, respectively, aiding in the identification of causal influences. Complex network analysis and dynamical systems theory contribute to the understanding of the structural and dynamical properties of networks, shedding light on the underlying causal mechanisms (Hartung et al., in preparation).

As the study of complex networks continues to evolve, new methods and approaches are emerging to tackle the challenges of inferring causality in ever-larger and more intricate systems. The integration of machine learning, data science, and domain expertise holds great promise for advancing our understanding of causation in complex networks. By leveraging these powerful tools and frameworks, we can unravel the intricate web of cause and effect, leading to new insights, predictions, and interventions in a wide range of scientific and real-world applications.

### Artificial intelligence to elucidate causation in complex networks

In the era of big data and advanced computational capabilities, AI has emerged as a transformative force in the study of complex networks. Complex networks, which are characterized by intricate patterns of interactions and dependencies, pose significant challenges for traditional analytical methods. However, AI techniques offer powerful tools for unraveling the causal relationships within these networks (Fig. 9), enabling to gain deeper insights into their structure, dynamics, and behavior.

Recent advancements in Natural Language Processing (NLP) have transformed the process from a manual bottleneck into an automated pipeline. By utilizing Named Entity Recognition (NER) and relationship extraction algorithms, we can now systematically mine vast repositories of unstructured text to identify causal links between molecular initiating events and adverse outcomes (Corradi et al. 2022). In their 2022 paper, in the context of the European ONTOX project (Vinken et al. 2021), they proposed that NLP could be used as a support tool for Bradford-Hill criteria suggesting that NLP could automatically gather weight-of-evidence to support the “*biological plausibility*” and “*empirical support*” criteria. For example, deep learning models have successfully reconstructed causal networks for complex liver pathologies like cholestasis and steatosis directly from scientific abstracts, creating *‘living’* systematic reviews that update as new data emerges (Corradi et al. 2024).

#### Data analysis and pattern recognition

One of the fundamental tasks in studying complex networks is to identify meaningful patterns and relationships within the vast amounts of data generated by these systems. AI techniques, such as machine learning and data mining, have proven invaluable in this regard. By leveraging advanced algorithms and statistical methods, AI can uncover hidden patterns and correlations that may indicate causal relationships between different nodes in the network.

For example, unsupervised learning techniques like clustering and dimensionality reduction can help group similar nodes together based on their attributes or behavior, revealing potential causal associations. Supervised learning methods, such as classification and regression, can be used to predict the state or behavior of nodes based on the states of other nodes, providing insights into the directionality and strength of causal influences.

#### Predictive modeling

AI-powered predictive modeling is another crucial tool in the study of causation in complex networks. By training machine learning models on historical data, we can develop accurate predictors of future network states and behaviors. These models can capture the complex non-linear relationships and dependencies within the network, enabling us to forecast the impact of interventions or perturbations on the system. Techniques such as time series analysis, recurrent neural networks (RNNs),^3^ and long short-term memory (LSTM) networks^4^ are particularly well-suited for modeling the temporal dynamics of complex networks. By incorporating information about the network’s structure and the states of individual nodes, these models can provide nuanced predictions of how the network will evolve over time, shedding light on the causal mechanisms at play.

#### Causal inference

Causal inference is at the heart of understanding causation in complex networks, and AI techniques have revolutionized this field. Traditional methods for causal inference, such as Regression Analysis, often face limitations when dealing with the scale and complexity of real-world networks. However, AI-based approaches, such as Bayesian networks and structural equation modeling, offer powerful alternatives for inferring causal relationships from observational data. Bayesian networks, for example, represent the probabilistic dependencies between variables in a network using directed acyclic graphs (DAG).^5^ By learning the structure and parameters of these networks from data, researchers can identify the most likely causal relationships and quantify the strength of these relationships. Similarly, structural equation modeling allows for the specification and testing of causal hypotheses by modeling the relationships between latent and observed variables.

#### Simulation and optimization

AI-driven simulation and optimization techniques play a crucial role in understanding the behavior of complex networks under different conditions. By building realistic computational models of networks and simulating their dynamics, researchers can explore the effects of various interventions and perturbations on the system. This allows for the identification of key causal factors and the evaluation of potential strategies for managing or optimizing network performance. Techniques such as agent-based modeling (Monti et al. 2023), reinforcement learning,^6^ and evolutionary algorithms^7^ can be used to simulate the behavior of individual nodes and their interactions within the network. These simulations can provide valuable insights into the emergent properties and collective dynamics of the system, revealing the underlying causal mechanisms that give rise to observed phenomena.

#### Network reconstruction

In many real-world scenarios, the complete structure of a complex network may be unknown or too complex to analyze directly. In such cases, AI techniques can be employed to reconstruct or infer the network structure from available data. This process, known as network reconstruction or network inference, involves identifying the most likely connections and dependencies between nodes based on their observed behaviors or attributes. Machine learning methods, such as graph neural networks (GNNs)^8^ and probabilistic graphical models (Koller and Friedman 2009), have shown great promise in this area. By learning the patterns and regularities in the data, these models can infer the underlying network structure and uncover hidden causal relationships. Network reconstruction is particularly valuable in fields like systems biology, where the goal is to infer gene regulatory networks from high-throughput omics data.

#### Deep learning for complex networks

Deep learning, a subset of AI that focuses on training deep neural networks, has emerged as a powerful tool for analyzing complex networks. In particular, graph neural networks (GNNs) have gained significant attention due to their ability to directly operate on data structured as graphs, which is the natural representation for many complex networks (Mobaraki and Khan 2023). GNNs can learn rich, hierarchical representations of network nodes and edges, capturing the complex non-linear relationships and dependencies within the network. By incorporating information about the network’s topology and node attributes, GNNs can perform tasks such as node classification, link prediction, and community detection with high accuracy. This makes them valuable tools for uncovering causal relationships and predicting the behavior of complex systems.

#### Understanding network dynamics

AI techniques are also instrumental in analyzing the dynamics of complex networks, i.e., how the network evolves and changes over time (Yu et al. 2004). By modeling the temporal patterns and dependencies within the network, AI can help identify the key drivers of network dynamics and predict future states of the system. Techniques such as dynamic Bayesian networks,^9^ temporal graph neural networks,^10^ and recurrent neural networks^3^ are particularly well-suited for capturing the temporal aspects of complex networks. These models can learn the patterns of node and edge evolution, detect anomalies or critical transitions, and forecast the long-term behavior of the network. Understanding network dynamics is crucial for identifying causal relationships that may be obscured by the static structure of the network.

#### Anomaly detection

Anomaly detection^11^ is another important application of AI in the context of complex networks. By learning the normal patterns and behaviors of a network, AI models can identify unusual or unexpected deviations that may indicate the presence of causal factors or interventions. Anomaly detection can be used to identify critical nodes or edges, detect cascading failures or vulnerabilities, and monitor the health and stability of the network. Techniques such as one-class classification,^12^ clustering,^13^ and autoencoders^14^ can be used to model the normal behavior of a network and flag anomalies that deviate from this behavior. By analyzing these anomalies and their potential causes, researchers can gain insights into the underlying causal mechanisms and develop strategies for mitigating risks and enhancing network resilience.

#### Conclusion

Artificial intelligence has emerged as a transformative force in the study of causation in complex networks. By leveraging advanced computational techniques and machine learning algorithms, AI enables researchers to uncover hidden patterns, infer causal relationships, and predict the behavior of complex systems. From data analysis and pattern recognition to causal inference and network reconstruction, AI provides a comprehensive toolkit for understanding the intricate web of cause and effect within networks.

As the scale and complexity of real-world networks continue to grow, the role of AI in studying causation will only become more crucial. By integrating AI with domain expertise and traditional analytical methods, researchers can gain unprecedented insights into the structure, dynamics, and behavior of complex systems. This knowledge will be invaluable for addressing pressing challenges in fields ranging from biology and medicine to social science and engineering.

However, it is important to recognize that AI is not a panacea for understanding causation in complex networks. Careful consideration must be given to the limitations and biases of AI models, as well as the need for interpretability and explainability in the results. Collaboration between AI experts and domain specialists is essential for ensuring the validity and reliability of the insights generated by AI techniques.

Table 1 gives examples of the use of these technologies in toxicology.

**Table 1:** Examples of complex-network causality methods used in toxicology

Method (Chapter 6)	What it’s been used for in toxicology	Example(s)
Causal graphs / DAGs	Making causal assumptions explicit (confounding, mediation, interventions) in environmental/cumulative risk contexts	Brewer et al. 2017 used DAGs to support causal inference in cumulative risk assessment
Bayesian networks / probabilistic graphical models	Integrating heterogeneous evidence streams into probabilistic hazard/potency conclusions; uncertainty propagation across a mechanistic network	Jaworska et al. 2010 proposed a Bayesian-network ITS framework under REACH. Jaworska et al. 2015 implemented a Bayesian ITS for skin sensitization potency (quantitative WoE + adaptive testing)
Probabilistic AOP / AOP networks (qAOP via BN/regression)	Quantifying key-event relationships and propagating uncertainty through AOP networks	Moe et al. 2021 quantified an AOP network using Bayesian regression + Bayesian network modeling. Spînu et al. 2022 built a probabilistic model for a DNT AOP network
Causal biological network models (signed cause–effect networks)	Mechanistic interpretation of tox ‘omics by mapping measured changes onto directed cause/effect networks	Martin et al. 2012 introduced Network Perturbation Amplitude using causal biological networks. (Applied/reviewed in systems toxicology by Hoeng et al. 2014.)
Causal reasoning / network contextualization from ‘omics	Inferring upstream drivers and causal paths from toxicogenomics data (often AOP-aligned)	Trairatphisan et al. 2021 applied CARNIVAL (causal network contextualization) to TG-GATEs data for drug-induced liver injury
Granger causality	Detecting/characterizing toxidromes or exposure signatures from time-lagged physiological coupling	Difrancesco et al. 2023 used Granger-causal structure between ECG/respiration features to help distinguish VX vs fentanyl exposure signatures
Transfer entropy (directed information flow)	Directed “*effective connectivity*” changes in neuronal networks under neurotoxicant exposure	Hiolski et al. 2016 quantified changes in network connectivity under domoic acid exposure using transfer entropy-based connectivity inference
Complex network analysis (graph theory on tox data)	Mapping many-to-many chemical–phenotype relationships (toxidromes) and discovering structural regularities	Bhavnani et al. 2007 performed network analysis of toxic chemicals vs. acute symptoms from the WISER database to inform first-responder decision support
Dynamical systems / tipping points	Identifying non-linear transitions where systems fail to recover after perturbation (dose/time “points of no return”)	Saili et al. 2020 characterized a toxicological tipping point during human stem cell differentiation under chemical perturbation
Dynamical systems (ODE-based qAOP modeling)	Time-course causal modeling of KEs/KERs to predict an AO; supports intervention/perturbation analysis	Di Tillio and Beltman 2024 proposed an ODE-based qAOP framework (incl. Bayesian inference / model updating concepts). (Broader qAOP context: Conolly et al. 2017.)
AI/NLP for causal evidence extraction (“*living*” mechanistic reviews)	Automated extraction of mechanistic relations (MIE → KE → AO) to support causal plausibility/WoE building	Corradi et al. 2022 reviewed NLP to delineate AOPs and guide NAMs. Corradi et al. 2024 demonstrated NLP extraction of mechanistic information for toxicology
Knowledge graphs + interpretable deep learning (mechanism-tracing explanations)	Explaining toxicity predictions by tracing paths through curated biological hierarchies/relations	Hao et al. 2023 (“AIDTox”) used a curated knowledge graph to provide mechanistic explanations for toxicity predictions

Looking ahead, the continued development and refinement of AI methods for studying complex networks holds immense promise. As new algorithms and architectures emerge, and as computational capabilities continue to grow, we can expect to see even more powerful tools for unraveling the mysteries of causation in complex systems. By harnessing the power of AI, researchers will be able to tackle ever-more-challenging questions and drive new discoveries that shape our understanding of the world around us.

## Explainable AI in neural networks and cross-fertilization with AOPs

**Fig. 10:**
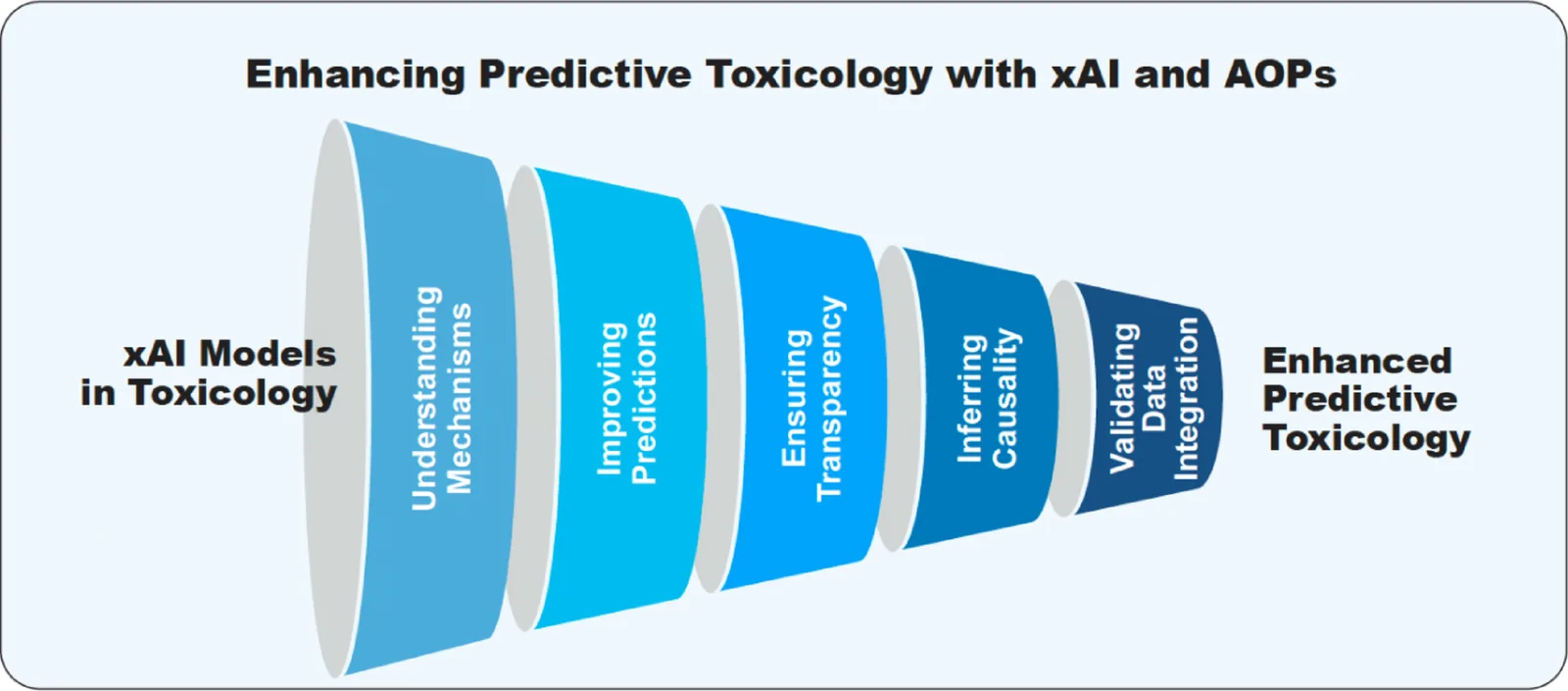
Enhancing predictive toxicology with explainable AI and adverse outcome pathways

AI has made significant strides in various domains, including toxicology. Neural networks, a subset of AI, have shown great promise in predicting toxicological outcomes. However, these models are often seen as “*black boxes*,” where the decision-making process is opaque. Explainable AI (“xAI”) (Linardatos et al. 2021; Sadeghi et al. 2024) has emerged as a field that aims to make the behavior of AI models transparent and understandable to humans (Zhou et al. 2023; Budhkar et al. 2025). In parallel, AOPs have gained traction in toxicology as a structured way to represent the mechanistic understanding of how exposure to a substance can lead to an adverse health outcome. This chapter explores the potential for cross-fertilization between xAI and AOPs, and how their integration can advance the field of predictive toxicology (Fig. 10).

### Understanding mechanisms

AOPs provide a framework to organize knowledge about toxicological mechanisms (Leist et al. 2017), from the initial molecular interaction of a toxicant with a biological system, through a series of key events, to an adverse outcome. xAI techniques can complement AOPs by helping to interpret complex neural network models that predict these pathways. For example, layer-wise relevance propagation (LRP) can highlight which input features (such as molecular descriptors or omics data) are most important for a model’s prediction of a key event or adverse outcome. This can provide insights into the biological mechanisms driving the model’s decisions, making them more understandable and trustworthy.

### Improving predictions

Neural networks have shown impressive performance in predicting toxicological outcomes from large datasets (Luechtefeld et al. 2018). However, without a clear understanding of how these predictions are made, it can be difficult to trust their outputs, especially in a regulatory context. xAI methods can help explain these predictions, ensuring that the models are reliable and their decisions are based on plausible biological mechanisms.

For instance, saliency maps (Simonyan et al. 2013) can visualize which parts of an input (such as regions of a chemical structure or subsets of genes) have the greatest influence on a model’s prediction. If these salient features align with known toxicological mechanisms, as described by AOPs, it increases confidence in the model’s predictions. Conversely, if the model is focusing on biologically implausible features, it suggests that the model may be overfitting or learning spurious correlations.

### Model transparency

A key challenge in applying AI to toxicology is ensuring that models are transparent and their decisions are interpretable. Regulators and decision-makers need to understand the basis for a model’s predictions in order to trust and act upon them. AOPs can help address this challenge by providing a biological framework for interpreting AI models. By grounding models in known biological mechanisms, AOPs can make them more credible and acceptable to stakeholders.

xAI techniques can further enhance the transparency of AI models by demystifying their decision-making process. For example, model-agnostic methods like SHAP (SHapley Additive exPlanations) (Lundberg and Lee 2017) can quantify the contribution of each input feature to a model’s output, providing a clear explanation of how the model arrived at its prediction. This can help build trust in the model and facilitate its adoption in regulatory and research settings.

### Causal inference

Establishing causal links between biological events is a central goal of AOPs. Understanding which key events are necessary and sufficient for an adverse outcome is critical for identifying opportunities for intervention and prevention. However, inferring causality from observational data is a challenge, and typically requires careful experimental design and manipulation of the system.

AI models, in conjunction with xAI techniques, can aid in causal inference by identifying which features or events are most predictive of an outcome. For example, if an XAI method consistently identifies a particular key event as being highly influential in predicting an adverse outcome, this suggests a potential causal link. This can guide further experimental investigation to confirm the causal relationship. Conversely, if an AI model can accurately predict an adverse outcome without relying on a presumed key event, it may call into question the necessity of that event in the causal chain.

### Data integration and validation

AOPs often integrate diverse types of data, from molecular and cellular assays to tissue and organ-level responses, to population-level outcomes. AI models, particularly deep learning approaches, are well-suited to handle this complexity and can learn to predict outcomes from high-dimensional, heterogeneous data.

However, the process of data integration itself can introduce additional opacity into the model. xAI can play a role here by ensuring that the integration process is transparent and that the resulting models are interpretable. For instance, if a model is integrating transcriptomic, proteomic, and metabolomic data to predict toxicity, xAI methods can reveal which data types and which specific features within each data type are driving the model’s predictions. This can help validate the data integration process and ensure that the model is leveraging biologically meaningful signals.

### Conclusion

The integration of xAI and AOP holds great promise for advancing the field of predictive toxicology. By leveraging the mechanistic framework of AOPs and the interpretability of xAI methods, we can develop AI models that are more transparent, trustworthy, and grounded in biological understanding. This synergy can lead to more reliable predictions of toxicological outcomes, clearer identification of causal mechanisms, and ultimately, safer and more efficient development of chemicals and drugs.

However, realizing this potential will require close collaboration between AI researchers, toxicologists, and regulatory scientists. It will be important to develop standardized approaches for representing AOPs in a machine-readable format, and for evaluating the biological plausibility of AI model explanations. Regulatory agencies will need to develop guidelines for the use of explainable AI in toxicological assessments, and to ensure that model transparency is maintained throughout the decision-making process.

As the fields of AI and toxicology continue to evolve, the integration of xAI and AOPs will be a key area of research and development. By working together, we can harness the power of AI to improve our understanding of toxicological mechanisms, and to protect human health and the environment.

## Practical applications of explainable AI and AOP in toxicology

The integration of xAI and AOP has the potential to revolutionize toxicology: By combining the predictive power of AI with the mechanistic understanding provided by AOPs, we can develop more efficient, reliable, and transparent approaches to assessing chemical safety. This chapter explores three key areas where this integration can have a practical impact: drug development, regulatory toxicology, and research.

### Drug development

The pharmaceutical industry is under constant pressure to bring new drugs to market quickly and safely (Meigs et al. 2018). However, the process of drug development is lengthy, expensive, and fraught with potential for failure. One of the key challenges is predicting potential adverse effects of new compounds early in the development process. This is where AI models, trained on large toxicological datasets, can play a crucial role.

By leveraging machine learning techniques, such as deep learning, we can build models that can predict the toxicity of new compounds based on their structural and physicochemical properties. These models can screen vast libraries of compounds virtually, identifying those that are likely to be safe and ruling out those with high toxicity potential. This can significantly accelerate the drug discovery process and reduce the number of animal tests required.

However, in order for these AI predictions to be trusted and actionable, they need to be explainable and biologically plausible. This is where AOPs come in. By mapping the predicted toxicities onto known Adverse Outcome Pathways, we can validate the AI model’s decisions and ensure they are grounded in mechanistic understanding. If the model predicts that a compound will cause liver toxicity, for example, we can check whether the key events in the relevant AOP (such as oxidative stress, inflammation, and cell death) are indeed being triggered by the compound.

xAI techniques, such as feature attribution methods, can further elucidate how the AI model arrived at its predictions. By highlighting which structural features of the compound are most responsible for its predicted toxicity, xAI can guide medicinal chemists in their efforts to optimize the compound’s safety profile.

### Regulatory toxicology

Regulatory agencies, such as the Environmental Protection Agency (EPA) and the European Chemicals Agency (ECHA), are tasked with assessing the safety of chemicals and making decisions about their use and disposal. These assessments are based on a combination of animal studies, in vitro tests, and computational models. AI has the potential to make these assessments more efficient and less reliant on animal testing.

By training AI models on large toxicological databases, such as ToxCast and Tox21, we can predict the potential hazards of new chemicals without the need for extensive animal studies. These models can be used to prioritize chemicals for further testing, focusing resources on those that are most likely to pose a risk.

However, for these AI models to be acceptable in a regulatory context, they need to be transparent and interpretable. Regulators need to understand how the model arrived at its predictions and what biological evidence supports them. xAI methods can provide this transparency, allowing regulators to peer inside the “*black box*” of the AI model and understand its decision-making process.

Moreover, by grounding AI predictions in AOPs, we can provide a clear mechanistic justification for regulatory decisions. If an AI model predicts that a chemical is likely to cause reproductive toxicity, for example, we can point to the specific key events in the relevant AOP that are being perturbed. This can help build confidence in the AI model’s predictions and facilitate their acceptance by regulatory bodies.

### Research

Beyond the practical applications in drug development and regulatory toxicology, the integration of xAI and AOPs can also drive fundamental research in toxicology. By applying AI techniques to large toxicological datasets, we can uncover new patterns and relationships that might not be apparent from traditional statistical analyses.

For example, unsupervised learning methods, such as clustering and dimensionality reduction, can help identify groups of chemicals that share similar toxicological profiles. By analyzing the structural and mechanistic features that these chemicals have in common, we can gain new insights into the underlying pathways of toxicity.

Moreover, AI models can suggest new hypotheses and point to potential new Adverse Outcome Pathways. If an AI model consistently predicts a certain toxicological outcome for chemicals with a specific structural feature, for instance, this could suggest a new molecular initiating event and the start of a new AOP. xAI methods can then be used to explore and validate these hypothesized pathways, guiding targeted experimental studies.

Finally, the integration of xAI and AOPs can facilitate the development of more predictive and biologically relevant in vitro models. By using xAI to identify the key molecular and cellular events that are most predictive of adverse outcomes, we can design in vitro assays that specifically target these events. This can lead to more efficient and reliable screening methods that reduce the need for animal testing.

### Importance of causality analysis for a human exposome project

Causality analysis is essential for the emerging Human Exposome Project (Hartung 2023, 2025; Sillé et al. 2024) because simply cataloguing thousands of exposures cannot reveal which ones actually drive disease. By tracing how real-world chemical, physical and psychosocial stressors perturb molecular networks and cascade through AOP, causal analytics turn descriptive exposome maps into actionable risk hierarchies. Systems-level methods combining network analysis with multi-omics and high-throughput assays help pinpoint “*key nodes*” whose perturbation propagates harm, and quantify how multiple exposures interact in time and dose to exceed resilience thresholds. Without this mechanistic lens, associations remain statistical shadows; with it, we can prioritize interventions, design targeted biomonitoring, and build predictive models that move public-health policy from hazard listing to prevention grounded in demonstrable cause-effect relationships.

### Conclusion

The integration of xAI and AOP has vast potential for practical application in toxicology. In drug development, it can accelerate the discovery of safe and effective new medicines. In regulatory toxicology, it can streamline risk assessment and provide clear mechanistic justifications for decisions. And in research, it can drive new discoveries and the development of more predictive testing methods.

However, realizing this potential will require close collaboration between AI experts, toxicologists, and regulatory scientists. It will be important to develop standardized approaches for integrating xAI and AOPs, and to validate these approaches in real-world case studies. Regulatory agencies will need to provide clear guidance on the use of AI in toxicological assessments, and to ensure that transparency and interpretability are maintained.

As the fields of AI and toxicology continue to advance, we can expect to see more and more examples of their successful integration. By harnessing the power of xAI and AOPs, we can make toxicology more efficient, more reliable, and more relevant to the protection of human health and the environment. This is an exciting frontier in toxicological science, and one that promises to have a profound impact on how we assess and manage chemical risks.

## Risk of Bias in the context of causation

Risk of bias (RoB), a critical aspect of study quality, refers to the systematic errors or deviations from the truth in results or inferences that can arise due to flaws in the design, conduct, analysis, or reporting of a study. This concept is central to evidence-based toxicology (EBT) and was covered in a parallel earlier paper (Hartung et al. 2025a). For the present article, three points suffice. First, every study entering a causality assessment must be screened for five classic domains, i.e., selection, performance/exposure, detection, attrition, and reporting bias, because systematic flaws in any one of them can distort apparent pathway strength or timing. Second, these domains should be screened with tools that match the evidence stream, for example, SYRCLE for animal experiments, OHAT for human and in‑vitro data, and the signaling questions in Hartung et al. (2025a) for computational or high‑throughput assays. Third, any study flagged “*high risk*” on a core domain might be down‑weighted or, for critical analyses, excluded, before GRADE scoring and Evidence‑to‑Decision (EtD) deliberation, ensuring that biased evidence does not over‑influence the network‑based causal model.

The assessment of RoB and the evaluation of causation in networked systems are interdependent and mutually informative. On one hand, the presence of certain types of bias, such as confounding or measurement bias, can lead to spurious or misleading conclusions about causation. On the other hand, a strong causal understanding of the networked system can help inform the design and interpretation of studies, and guide the assessment of RoB.

The complexity of biological systems poses challenges for both the assessment of risk of bias and the evaluation of causation presenting several unique considerations:

1. Confounding: Assessing the risk of confounding bias in networked systems requires a deep understanding of the underlying biology and the potential for complex interactions.
2. Measurement bias: Networked systems often involve measurements at multiple levels of biological organization, from molecular and cellular to tissue and organ levels. The choice of measurement methods and endpoints can introduce bias if they do not adequately capture the relevant aspects of the network.
3. Reporting bias: The complexity of networked systems may also increase the potential for selective reporting of results. With multiple pathways and endpoints to choose from, researchers may be tempted to focus on the most significant or interesting findings, while downplaying or omitting negative or inconsistent results.

The network-based, dynamic, multi-scale and quantitative perspective offered by systems toxicology (Hartung et al. 2017) is very powerful for dissecting the complex causal chains underlying toxicity pathways (Kleensang et al. 2014). The integration of computational modeling with multi-omics data provides an unprecedented platform for exploring causality in real-world biological complexity. Understanding how these concepts intersect and inform each other is crucial for advancing the field and improving the reliability and relevance of toxicological evidence.

## Toward a next‑generation hybrid framework for causation in EBT

**Fig. 11:**
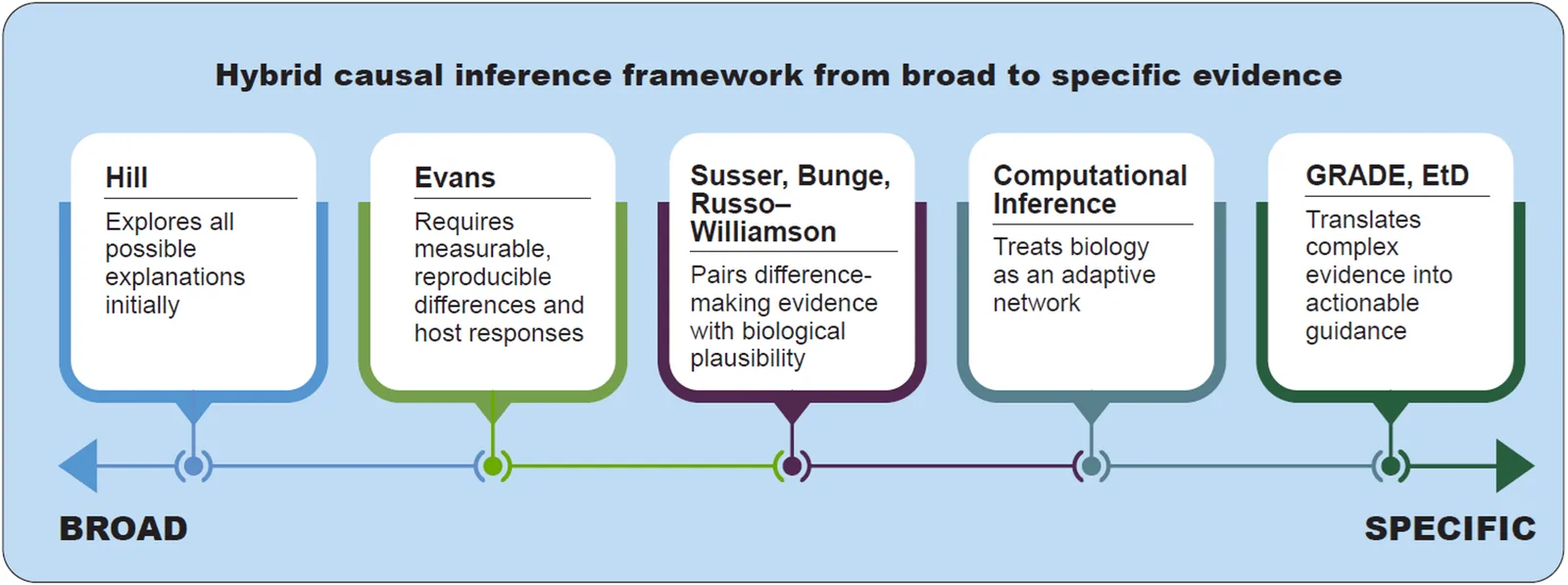
Hybrid causal inference framework from broad to specific evidence

Below we distill the diverse causal concepts reviewed in this article into an operational framework that toxicologists can apply across study types, from single‑cell assays to human epidemiology. The framework is organized around five complementary pillars that map directly onto the bullet points proposed (Fig. 11, Table 2).

**Table 2:** Hybrid framework for causation

Pillar	Core Idea	Practical Implementation	Key Outputs
1. Conceptual Breadth (Hill)	Start broad: explore *all* possible explanations before narrowing Approach of Human Exposome Project	Map the universe of exposures, outcomes, and modifiers using scoping reviews and text‑mining Systematic Review for event-event relationships Use Hill’s nine considerations as an initial lens for hypothesis generation rather than as pass/fail tests	Causal concept map; list of candidate pathways “*exposure pathway hypothesis*” & data gaps
2. Quantitative & Biological Specificity (Evans)	Require measurable, reproducible differences and explicit host responses	Specify a priori prevalence/incidence contrasts and dose–response gradients Embed mechanistic assays (omics, cell‑based PoT read‑outs) alongside population metrics Perform intervention or depletion studies	Effect estimates with confidence intervals; validated key events with quantitative relationships (KERs)
3. Mechanistic & Predictive Standards (Susser, Bunge, Russo–Williamson)	Pair difference‑making evidence with biological plausibility	Construct Directed Acyclic Graphs (DAGs) or Structural Equation Models that formalize competing hypotheses (Susser) Check *temporal priority* and *invariance* across contexts (Bunge) Demand *both* statistical association and confirmed pathway perturbation (Russo–Williamson)	Causal DAG with annotated evidence strength; consistency score across contexts
4. Network Science & Computational Inference	Treat biology as an adaptive network, not a linear chain Network analysis for consistency and effect modification	Build multi‑layer networks (gene, protein, cell, organ) and analyse centrality, controllability, attractor states Apply Bayesian network learning, graph neural networks, and causal discovery algorithms (e.g., NOTEARS, PCMCI) • Use in silico perturbation (virtual knock‑outs) to predict outcomes and identify tipping points	Network heat maps of perturbation amplitude; ranked list of causal hubs & emergent properties
5. Evidence Synthesis & Decision (GRADE, EtD)	Translate complex evidence into actionable guidance	• Grade certainty separately for difference‑making and mechanistic strands, then integrate (dual‑GRADE) • Populate EtD tables that weigh benefits, harms, values, feasibility, and equity • Express residual uncertainty quantitatively (probabilistic risk ranges) and qualitatively (confidence narratives)	Integrated EtD statement; probabilistic risk estimate; transparent audit trail

## Operational Workflow:

1. *Scoping & Question Formulation*—Define population, exposure, outcome, context; draft initial DAG (Hill scope).
2. *Systematic Evidence Retrieval*—Register protocol; harvest data across in vivo, in vitro, in silico, and human studies.
3. *Bias Appraisal & Data Extraction*—Apply SYRCLE,^15^ OHAT^16^ and other RoB‑Tox tools (Hartung et al. 2025a); record mechanistic relevance tags.
4. *Quantitative Synthesis*—Meta‑analyze prevalence/incidence (Evans) and dose–response; calibrate Bayesian priors.
5. *Mechanistic Integration*—Map key events onto AOP/AON (Adverse Outcome Network); validate with perturbation assays (Maertens et al. 2025).
6. *Network Modelling & Causal Discovery*—Fit computational models; test counterfactuals; estimate causal effect sizes.
7. *GRADE & EtD Translation*—Dual‑grade evidence, complete EtD; formulate recommendation with uncertainty bounds.

**Worked Example**: Imagine assessing whether Chemical X causes developmental neurotoxicity (DNT). A Hill-style scoping exercise first frames a PECO question and maps competing explanations (co-exposures, nutrition, maternal illness), while checking that temporality is plausible (maternal exposure during sensitive neurodevelopmental windows precedes the malformations) and that the association is not obviously inconsistent across study designs. Next, Evans-type operational checks quantify “*difference-making*” evidence: across eligible studies, exposed litters show a ~ threefold higher malformation rate (with attention to dose/response, replication, and reversibility), after screening key studies for risk of bias and extracting effect estimates suitable for synthesis. To make assumptions explicit and testable, a directed acyclic graph (DAG) is constructed that encodes the hypothesized causal structure and highlights likely confounders and mediators; in this scenario, the most coherent causal route implicates thyroid hormone disruption as an upstream driver of neurodevelopmental impairment. Mechanistic integration then moves from a single AOP to an AOP network, where network modeling (e.g., centrality/controllability) identifies deiodinase inhibition as a plausible hub event that could propagate downstream perturbations across multiple neurodevelopmental processes. Targeted assays are then selected to “*stress-test*” this hypothesis: biochemical confirmation of deiodinase inhibition, thyroid receptor pathway activity, and TH-dependent neurodevelopmental endpoints in fit-for-purpose NAMs (supported by omics signatures mapped onto the AOP network) provide evidence for key-event activation and essentiality. Evidence synthesis follows a dual-strand logic aligned to the Russo-Williamson idea: a dual-GRADE assessment rates the mechanistic strand as high certainty (coherent pathway mapping, consistent key-event concordance, and experimental perturbation evidence) while the difference-making strand remains moderate (e.g., residual indirectness and limitations typical of animal or observational evidence). Finally, an Evidence-to-Decision (EtD) table makes the normative step transparent: given the severity of the endpoint and a disproportionate burden on vulnerable populations (e.g., pregnancy/early life, populations with higher baseline susceptibility), the balance of consequences supports a precautionary regulatory restriction (or stringent exposure limits with targeted risk management), with residual uncertainty clearly documented.

In conclusion, a causation framework fit for 21st‑century toxicology must be polyglot: fluent in classical epidemiology, molecular mechanism, quantitative modelling, and decision science. By nesting Hill’s breadth inside Evans‑style rigor, overlaying Russo–Williamson dual evidence, leveraging network analytics, and sealing it with GRADE/EtD transparency, the proposed hybrid delivers a transparent, mechanistically grounded, and decision‑ready pathway from data to action. The evolution of toxicology toward human-relevant, mechanistically anchored, and reproducible methods necessitates a reevaluation of causal inference frameworks. While the Bradford Hill criteria laid the foundation, Evans’ postulates and more recent advancements such as Rothman’s sufficient-component cause model, the Russo-Williamson thesis, and network causal modeling, offer a more comprehensive and empirically robust strategy. With the advent of agentic AI,^17^ these complex analyses can be supported and increasingly independently carried out by pretrained and fine-tuned foundational models orchestrated in an agentic AI process (Luechtefeld and Hartung 2025). Agentic AI can accomplish a specific goal with limited supervision. It consists of AI agents, i.e., machine learning models that mimic human decision-making to solve problems in real time. In agent AI, each agent performs a specific subtask required to reach the goal and their efforts are coordinated through agent orchestration.

Such an integrative approach provides a scalable and adaptable model for causal inference across diverse toxicological contexts, ensuring the scientific rigor and human relevance necessary for 21st-century decision-making.

## Conclusion

The assessment of risk of bias (Hartung et al. 2025a, b) and the evaluation of causation in networked systems (here) are critical and interrelated challenges for evidence-based toxicology. By understanding the unique considerations and opportunities presented by networked systems, and by developing and applying new approaches and methods that integrate both perspectives, we can advance the field and improve the quality and impact of toxicological evidence. This will require a sustained and collaborative effort from the toxicology community, but the potential benefits for human and environmental health are substantial.

In conclusion, the synergy between causality analysis in networked systems, particularly through the lens of AOPs, and explainable AI in neural networks holds immense potential. This cross-fertilization can enhance our understanding of toxicological mechanisms, improve predictive models, and ensure that these models are interpretable and grounded in biological reality. The integration of these fields promises to advance both toxicological research and AI, leading to more effective and safer chemical and pharmaceutical developments. Overall conclusions:

1. Causality analysis in toxicology is crucial for building mechanistic understanding and confidence in safety assessments, but traditional approaches like the Bradford Hill criteria need to be adapted and extended to better handle the complexity of networked biological systems.
2. The development and application of AOPs provides a structured framework for organizing and integrating evidence of causal relationships across different levels of biological organization, but fully elucidating the multifactorial and nonlinear causal pathways within these networks requires the integration of computational modeling, multi-omics data, and high-throughput experimentation.
3. The synergy between AOPs and xAI methods holds great promise for enhancing the interpretability, biological plausibility, and regulatory acceptance of predictive toxicological models, with practical applications in drug development, hazard assessment, and mechanistic research.
4. Assessing the risk of bias is a critical component of evidence-based toxicology, but the unique characteristics of networked systems, such as the potential for confounding, measurement bias, and selective reporting, pose challenges for existing risk of bias assessment tools and require the development of more holistic and integrated approaches.
5. Advancing causality analysis and xAI methods in toxicology will require a sustained and collaborative effort from the toxicology, systems biology, data science, and regulatory communities, but the potential benefits for improving the quality and efficiency of chemical safety assessments and for protecting human and environmental health are substantial.

## Data Availability

No novel data was produced for this manuscript.

## Footnotes

1 https://www.gradeworkinggroup.org

2 https://www.ebtox.org

3 https://www.ibm.com/think/topics/recurrent-neural-networks

4 https://www.analyticsvidhya.com/blog/2021/03/introduction-to-long-short-term-memory-lstm/

5 https://www.ibm.com/think/topics/directed-acyclic-graph

6 https://www.ibm.com/think/topics/reinforcement-learning

7 https://www.sciencedirect.com/topics/mathematics/evolutionary-algorithm

8 https://www.ibm.com/think/topics/graph-neural-network

9 https://www.geeksforgeeks.org/artificial-intelligence/dynamic-bayesian-networks-dbns/

10 https://towardsdatascience.com/temporal-graph-learning-in-2024-feaa9371b8e2/

11 https://www.ibm.com/think/topics/anomaly-detection

12 https://soulpageit.com/ai-glossary/one-class-classification-explained/

13 https://venturebeat.com/ai/what-is-artificial-intelligence-ai-clustering-how-it-identifies-patterns/

14 https://www.ibm.com/think/topics/autoencoder

15 https://www.syrcle.network

16 https://ntp.niehs.nih.gov/research/assessments/noncancer/riskbias

17 https://www.ibm.com/think/topics/agentic-ai
